# Early Recognition and Referral of Acute Stroke in Primary and Emergency Care: A Systematic Review

**DOI:** 10.5811/westjem.50827

**Published:** 2026-01-24

**Authors:** Thamer Majed Almunif, Abdulaziz Fahd Alkaabba, Khaled Waleed Alomran, Abdullah Mfwadh Alanazi, Faris Nashmi Alharbi, Safar Saad Alshahrani, Naif Mansour Alsaeed, Rayan Ahmed Alabdulkader, Sultan Adel Alibraheem, Khaled Saeed Alzahrani, Mohammed Hamad Albagieh

**Affiliations:** College of Medicine, Imam Muhammad Ibn Saud Islamic University, Department of Family Medicine, Riyadh, Saudi Arabia

## Abstract

**Introduction:**

Early recognition and referral are critical to minimizing morbidity and mortality in acute stroke, but evaluation and referral processes differ worldwide. In this systematic review we examined the accuracy of recognition tools, referral patterns, outcomes, and factors affecting efficiency in primary and emergency care settings.

**Methods:**

Following PRISMA 2020 guidelines, we searched PubMed, Scopus, Web of Science, and Cochrane Library for studies published January 2003–December 2025. Eligible studies included randomized controlled trials, cohort, case-control, cross-sectional, and large case series (> 30 patients) involving adults with acute ischemic or hemorrhagic stroke. Risk of bias was assessed using Cochrane Risk-of-Bias 2 (RoB) and RoB in non-standardized studies-I. We extracted data on diagnostic accuracy, referral pathways, outcomes, and systemic factors.

**Results:**

We identified 206 papers, of which 33 studies met our inclusion criteria. Recognition tools such as Face, Arms, Speech, Time (FAST); Recognition of Stroke in the Emergency Room, the Cincinnati Prehospital Stroke Scale, and National Institutes of Health Stroke Scale showed good pooled sensitivity (79–95%) but variable specificity (52–84%). Newer technologies, including the PreHospital Ambulance Stroke Test, FAST-ED, and artificial intelligence (AI)-based models, showed promise but need validation. Referral strategies such as emergency medical services prenotification, dispatcher triage, and mobile stroke units reduced prehospital delays. Seven studies reported onset-to-door times 12–22 minutes faster and 7–12% increase in reperfusion eligibility. Increased referral efficiency was associated with a reduction in mortality of approximately 8–12% and improvements in functional independence of 10–15%, with persistent disparities reported in resource-limited settings.

**Conclusion:**

Early recognition and referral improve outcomes in patients with acute stroke. Structured tools and system-level interventions reduce mortality, while AI and mobile stroke units show promise. Strengthening referral systems and adopting cost-effective triage strategies may support equitable implementation, particularly in low-resource settings, as addressing systemic and geographic barriers is critical for equitable stroke care.

## INTRODUCTION

Stroke remains one of the leading causes of death and long-term disability globally, creating major healthcare and socioeconomic burdens. One in four adults may experience a stroke, with most cases occurring in low- and middle-income countries, although high-income countries also face challenges from aging populations and risk factors such as hypertension, diabetes, and obesity.^1^ Despite prevention and treatment advances, outcomes depend heavily on time from symptom onset to treatment. During untreated cerebral ischemia, millions of neurons are lost every minute.^2^ Survivors often face long-term disabilities that reduce quality of life and place heavy demands on families and healthcare systems, making early recognition and management a public health priority.

Acute stroke treatment is highly time sensitive. Reperfusion therapies such as intravenous thrombolysis and endovascular thrombectomy are most effective when delivered quickly, ideally within 4.5 hours for thrombolysis and up to 24 hours for thrombectomy.^3^ Any delays in recognition or referral can exclude patients and increase mortality.^4^ International guidelines emphasize rapid triage, standardized tools, and optimized referral pathways.^5^ However, delays in early stages of care remain a barrier.

Primary and emergency care professionals are often first to encounter patients. Their ability to recognize and respond promptly determines hospital arrival and eligibility for reperfusion.^6^ Tools such as the Face Arm Speech Time (FAST) test, Recognition of Stroke in the Emergency Room (ROSIER) scale, Cincinnati Prehospital Stroke Scale (CPSS), and National Institutes of Health Stroke Scale (NIHSS) improve detection but vary in accuracy. The FAST test shows sensitivity of 94–95% but specificity of 55–60%,^9^ reflecting a trade-off between early detection and false-positive activation due to stroke mimics such as seizures or hypoglycemia. While the ROSIER and CPSS scales can identify most strokes, they similarly risk false positives.^7^ Advanced neuroimaging remains the diagnostic gold standard, although decisions often occur before imaging in resource-limited settings.

System-level barriers persist. Access to imaging and stroke-ready hospitals is inequitable, especially in rural areas.^8^ Organizational obstacles include unreliable emergency medical services (EMS) triage and inconsistent prenotification, while clinician barriers involve variable training.^9^ Patient-level challenges include misdiagnosis, reported in 10–25% of cases,^10^ as well as delays from long transport, limited stroke-unit capacity, and urban–rural disparities.^11^

Referral patterns significantly affect outcomes. Use of EMS prenotification and priority dispatch reduces prehospital delays and increases eligibility for reperfusion; EMS prenotification and dispatcher-supported triage have been associated with reductions in onset-to-door times of approximately 12–22 minutes and increases in reperfusion eligibility of 7–12%.^12^ Conversely, missed recognition increases door-to-needle times. Recognition by EMS has been linked with shorter delays and higher reperfusion rates.^13^ Emerging technologies also hold promise. Artificial intelligence-based tools using video, audio, or magnetic resonance imaging (MRI) analysis report sensitivities > 79% and specificities > 84%.^14^ Machine-learning applied to hemodynamic signals and biomarkers may further aid triage.^15^ While early in development, such tools could complement existing recognition systems.

**Population Health Research Capsule**

What do we already know about this issue?

*Delayed recognition and referral of acute stroke reduces eligibility for reperfusion therapy and worsens mortality and functional outcomes*.

What was the research question?

*How accurate are stroke recognition tools, and how do referral pathways affect outcomes in primary and emergency care?*

What was the major finding of the study?

*Across 33 studies, early recognition and referral reduced delays by 12–22 minutes and mortality by 8–12%*.

How does this improve population health?

*Improving early stroke recognition and referral systems can reduce preventable deaths and disability, especially in resource-limited settings*.

Although many studies address diagnostic tools or hospital care, few reviews examine referral pathways across both primary and emergency care settings. Existing reviews often focus narrowly on single tools or detection methods without linking recognition, referral, and outcomes. Furthermore, previous reviews have not integrated diagnostic accuracy with real-world referral patterns and patient outcomes across diverse healthcare systems, restricting implementation of evidence-based strategies. Our objective in this study was to systematically evaluate diagnostic accuracy, referral patterns, and outcomes associated with early recognition and management of acute stroke in primary and emergency care between 2003–2025.

## METHODS

### Study Design

This systematic review followed the 2020 Preferred Reporting Items for Systematic Reviews and Meta-Analyses (PRISMA) guidelines^16^ and synthesized evidence on early recognition and management of acute stroke in primary and emergency care. Owing to heterogeneity in study designs, populations, and outcomes, we summarized findings narratively rather than through meta-analysis. Heterogeneity was assessed using qualitative comparison across study designs, populations, and outcome definitions; statistical measures such as I^2^ were not feasible due to inconsistent reporting of accuracy metrics. We monitored inter-rater agreement during screening and resolved discrepancies by consensus.

### Eligibility Criteria

We included studies published between January 2003–December 2025 involving adults with acute ischemic or hemorrhagic stroke in primary or emergency care settings. Eligible designs were randomized controlled trials, cohort, case-control, cross-sectional, and case series (≥ 30 participants). To be included, studies had to report diagnostic accuracy, referral patterns, or patient outcomes. We excluded systematic reviews, meta-analyses, editorials, letters, case reports (< 30 patients), animal studies, papers that were not published in English, and those without relevant outcome data.

### Information Sources and Search Strategy

We performed searches in PubMed, Embase, Scopus, Web of Science, and Cochrane Library, supplemented by World Health Organization (WHO) and major stroke organization reports. The PubMed strategy combined MeSH and free-text terms for stroke, recognition tools, primary/emergency care, referral, and outcomes, limited to 2003–2025. This strategy was adapted for the other databases. Full search strategies for each database are available in the Appendix.

### Study Selection and Data Extraction

Two reviewers independently screened titles, abstracts, and full texts, resolving disagreements by consensus or a third reviewer. The process is detailed in the PRISMA flow diagram (Figure). Data were extracted using a standardized form, including study details (author, year, country, setting), design, population (sample size, age, sex, stroke type), recognition tools (FAST, ROSIER, NIHSS, CPSS, imaging), referral patterns (time, facility, protocols), and outcomes (mortality, disability, quality of life, time to treatment). Barriers and facilitators at systemic, organizational, or clinician levels were also captured.

### Risk of Bias Assessment

We assessed the 33 included studies with the Cochrane Risk of Bias (RoB) 2 tool for randomized trials^17^ and RoB in non-randomized studies-I for observational designs.^18^ Assessments were conducted independently by two reviewers, with disagreements resolved by consensus or arbitration. These evaluations informed the interpretation of findings.

### Data Synthesis

Narrative synthesis summarized diagnostic accuracy, referral patterns, and outcomes. Meta-analysis of sensitivity and specificity was considered but deemed inappropriate due to substantial heterogeneity in study populations, tools, and reporting. Differences in reference standards (computed tomography [CT] vs MRI), outcome definitions, cutoff thresholds, and inconsistent reporting of specificity values further prevented quantitative pooling. Studies reporting both sensitivity and specificity of recognition tools were synthesized descriptively, with full values available in Table 1.

## RESULTS

This review synthesized evidence from 33 studies (2003–2025) on diagnostic accuracy, referral patterns, and outcomes of early recognition and management of acute stroke in primary and emergency care. Results are presented as study selection, study characteristics, RoB, diagnostic accuracy, referral patterns, patient outcomes, and barriers/facilitators.

### Study Selection

The initial search yielded 541 records; 206 remained after duplicates. Screening excluded 135 (65.5%), leaving 71 for full-text review. We excluded 38 studies (one irrelevant, 35 ineligible, two wrong population). Thirty-three studies were finally included. (See PRISMA diagram, Figure.)

### Characteristics of Included Studies

The 33 studies were published between 2003–2025 across Europe, North America, Asia, and the Middle East, with designs including randomized trials, cohort, case-control, and cross-sectional studies. Sample sizes ranged from <100 to > 4,000 patients. Populations were mostly middle-aged to elderly, with a slight male predominance (52–60% male across most studies). Ischemic stroke was most frequently studied, although hemorrhagic stroke and transient ischemic attacks were also represented. Recognition strategies included FAST, ROSIER, CPSS, NIHSS, and other prehospital tools. Computed tomography and MRI were used in nearly all studies for confirmation or outcome assessment. Outcomes reported covered diagnostic accuracy, referral times, treatment eligibility, mortality, disability, and functional independence. Some studies also assessed system-level factors such as EMS training and prehospital notification. Key details are summarized in Table 2 and Table 3.

### Risk of Bias Assessment

Seven studies were randomized controlled trials (RCT); the rest were observational. The RCTs generally showed low risk of bias in randomization and outcome measurement, although allocation concealment and blinding were occasional concerns. Observational studies often had moderate risk due to confounding, selection bias, and incomplete reporting. No study was excluded, but results from moderate/serious risk studies were interpreted cautiously (see Table 4 and Table 5).

### Diagnostic Accuracy of Recognition Tools

We evaluated recognition strategies in all studies, although quantitative accuracy data were inconsistent. The FAST, ROSIER, CPSS, and NIHSS scales were most frequently assessed. Sensitivities ranged 79–95%, with specificities 52–84%. The FAST and CPSS tests were reliable for EMS use, while ROSIER performed well in large diagnostic studies. The NIHSS, used mainly in emergency departments, improved detection of large vessel occlusions but was less practical for prehospital use. Novel methods such as PreHAST, FAST-ED, and AI-based tools reported sensitivities of 66–79% with variable specificities. Machine-learning models using hemodynamic biomarkers (eg, blood pressure variability, pulse waveform features) and serum biomarkers (eg, D-dimer, S100 calcium-binding protein B, and glial fibrillary acidic protein) showed early promise but lacked large-scale validation. Findings are summarized in Table 6.

### Referral Patterns

Timely referral was central across studies. Structured dispatcher systems like the Danish Index and the Rapid Emergency Triage and Treatment System (RETTS) improved referral efficiency, reducing prehospital delays and under-triage.^19^ Across studies, dispatcher-based systems reduced onset-to-door times by approximately 12–22 minutes. mobile stroke units, notably the **Stroke-Einsatz-mobile** stroke unit (STEMO) system, reduced time to imaging and improved eligibility for reperfusion.^20^ Reported reductions ranged from 8–17 minutes to CT, with corresponding increases in reperfusion eligibility of 7–12%.

Training and protocol interventions produced mixed results. The Paramedic Acute Stroke Treatment Assessment (PASTA) trial found no significant delay reduction with enhanced paramedic training,^21^ while the prehospital acute stroke severity toll (PreHAST) integration improved recognition of large vessel occlusion and expedited referral.^22^ The PreHAST-based EMS triage improved detection of large vessel occlusion by 9–15% and shortened referral intervals by 5–12 minutes. Persistent barriers included delays in secondary transfers, rural access challenges, and variability in dispatcher accuracy. Referral outcomes are summarized in Table 7.

### Subgroup Analyses

By setting, nine primary care studies reported moderate sensitivity (70–90%) but frequent referral delays beyond the thrombolysis window. Twenty-four emergency care studies, especially those using dispatcher protocols and mobile stroke units, showed shorter delays and higher treatment eligibility. Dispatcher-supported emergency care studies reported referral acceleration of 12–20 minutes.

By region, 14 European studies had structured prehospital systems, including dispatcher tools and mobile stroke units, reducing delays. Five studies from the Middle East and seven from Asia cited limited imaging and variable EMS training, while seven North American studies (more often tested AI-assisted tools with improved specificity). Regional differences reflected system capacity, with Middle East/Asia studies reporting less imaging availability and EMS training variability.

By recognition tool, traditional scales (FAST, CPSS, ROSIER) showed high sensitivity (80–95%) but low specificity (50–65%), leading to false positives. Models based in AI demonstrated higher specificity (> 75%) but lacked validation. The NIHSS provided detailed assessments but was less practical for prehospital use (Table 8).

### Patient Outcomes

Improved recognition and referral consistently correlated with better outcomes. The EMS prenotification, dispatcher triage, and mobile stroke units shortened door-to-needle times and increased reperfusion rates, leading to higher independence. For example, STEMO patients had higher thrombolysis rates and more often achieved modified Rankin scale ≤ 2 at 90 days.^23^ Across studies, mobile stroke unit-supported pathways reduced door-to-needle times by 8–17 minutes and increased reperfusion eligibility by 7–12%. Use of the Danish Index similarly reduced mortality.^24^ Studies reported mortality reductions of approximately 8–12% when dispatcher-supported recognition systems were used.

Scales like PreHAST, FAST-ED, and PASS identified large vessel occlusions effectively. PreHAST improved referral and outcomes, while PASS showed modest benefit due to paramedic variability.^12^ The PreHAST-based triage improved detection of large vessel occlusion by 9–15% and modestly reduced referral delays by 5–12 minutes. Despite these gains, disparities persisted in rural and resource-poor settings, where delayed transport and limited stroke unit access were linked with worse outcomes. The PASTA trial showed no functional outcome benefit despite protocol training,^24^ underscoring the gap between structured interventions and patient benefit.

Overall, early recognition and efficient referral improved mortality and morbidity, although benefits were lower in rural/underserved regions, where delayed transport and imaging shortages were associated with poorer functional outcomes.

### Barriers and Facilitators

Barriers operated at system, organizational, and patient levels. System-level barriers included rural location, limited stroke centers, absence of mobile stroke units, and transfer delays. Organizational factors such as dispatcher accuracy and inconsistent EMS triage also hindered care. Clinician-level barriers included differences in training and protocol adherence.^22^ At the patient level, delays were linked to poor symptom awareness, atypical presentations, or reluctance to seek care. Public education improved awareness but had limited impact on treatment timelines. Studies noted that public education campaigns increased stroke symptom recognition by 10–18% but did not translate into equivalent reductions in onset-to-door times.

Facilitators included EMS training, structured protocols, and emerging technologies. Artificial intelligence tools offered decision support, particularly in resource-limited areas. Mobile stroke units enabled rapid imaging and treatment initiation but were costly to scale.

## DISCUSSION

This systematic review synthesized 33 studies (2003–2025) examining early recognition and management of acute stroke in primary and emergency care. The findings highlight the role of structured recognition tools, efficient referral systems, and system-level support in improving patient outcomes.

### Summary of Key Findings

Widely used tools such as FAST, ROSIER, CPSS, and NIHSS consistently showed high sensitivity but variable specificity.^19^,^22^,^23^ Reported sensitivities exceeded 80%, supporting their use as first-line screening methods. Promising approaches included AI-based models and newer triage tools such as FAST-ED and PreHAST, although validation remains limited. Among traditional scales, FAST and CPSS were the most consistently reliable for EMS settings, while PreHAST showed the strongest potential for triage focused on large vessel occlusion.

Referral systems strongly influenced outcomes. Structured dispatcher frameworks, including the Danish Index^19^,^24^ and RETTS, improved prioritization and timely access to reperfusion therapy. Mobile atroke units, such as STEMO, provided immediate imaging and treatment initiation, significantly improving functional outcomes.^20^ However, training interventions alone, such as the PASTA trial, showed limited impact without broader system-level changes.

Improved referral efficiency consistently reduced mortality and increased functional independence (modified Rankin scale ≤ 2). Yet rural and resource-limited settings faced persistent delays, limited access to stroke centers, and poorer outcomes. Barriers persist despite advances. Heterogeneity in study design and reporting prevented quantitative meta-analysis, requiring narrative synthesis. Geographic disparities remain significant, with rural and low-resource settings facing delays in imaging, workforce shortages, and limited EMS infrastructure. Such inequities are most pronounced in low- and middle-income countries.

### Interpretation of Findings

These results reinforce global priorities emphasizing time-sensitive stroke care. Recognition tools are effective screening measures, but variable specificity highlights the need for confirmatory imaging and careful balance between over-triage and missed diagnoses. Heterogeneity in reporting precluded pooled meta-analysis, necessitating narrative synthesis.

Referral strategies clearly improve timeliness and treatment eligibility. Mobile stroke units represent a valuable but resource-intensive model suited to urban, high-income settings.^20^ The AI-based diagnostics are attractive where trained personnel are scarce, although rigorous validation is still required. Persistent system and patient-level barriers indicate that recognition tools and referral systems alone are insufficient. Public awareness campaigns improved symptom recognition but had little effect on treatment delays. Alignment with established global frameworks such as WHO’s Global Stroke Roadmap and American Stroke Association (ASA) guidance could help standardize early recognition and prehospital referral strategies across diverse health systems. Global frameworks, including the WHO Global Action Plan for Noncommunicable Diseases (2013–2030) and the WHO Global Stroke Action Plan (2016–2030), highlight opportunities to adapt local strategies. Practical approaches such as community-based triage, dispatcher-assisted algorithms, and mobile health apps could expand early recognition and referral options at lower cost. Alignment with ASA and WHO early stroke management recommendations may further support harmonized referral pathways across diverse systems.

### Strengths and Limitations

Strengths of this review include a comprehensive synthesis across regions and two decades, use of validated risk-of-bias tools, and inclusion of both traditional and innovative diagnostic approaches. Limitations include inconsistent reporting of accuracy metrics, heterogeneity in design and context, and reliance on narrative rather than quantitative synthesis. Most studies originated from high-income countries, limiting generalizability to low- and middle-income settings.

### Implications for Practice and Research

The evidence supports embedding structured recognition tools, EMS prenotification, and dispatcher triage into routine prehospital and emergency systems.^19^ Traditional scales such as FAST demonstrated high sensitivity but modest specificity, while AI-based approaches showed variable but promising accuracy (Table 8). Future research should validate AI diagnostics and evaluate the scalability of mobile stroke units^20^ in diverse contexts. Future research should prioritize multicenter validation trials of both traditional and AI-based recognition tools, with emphasis on scalability, cost-effectiveness, and applicability in diverse contexts. Evaluating mobile health applications and mobile stroke units across different socioeconomic settings will be vital. In parallel, implementation studies focusing on equity, feasibility, and integration into national stroke pathways are needed to guide real-world adoption. Developing a flexible set of service models as part of national and regional protocols could reduce inequalities in access to reperfusion therapy and support more equitable global stroke care. Integrating referral protocols with national and international frameworks (eg, WHO, ASA) may help reduce variability in practice and ensure standardized care pathways.

In low- and middle-income, mobile stroke units and advanced imaging are often unrealistic. Pragmatic strategies include structured EMS and community health worker training, simplified screening tools (FAST, PreHAST), telemedicine consultation with neurologists, and referral networks leveraging mobile technology. These incremental measures could improve timeliness and outcomes despite limited infrastructure.

This review underscores that early recognition and efficient referral improve survival and functional outcomes after stroke. Structured tools, dispatcher systems, and mobile stroke units are effective in high-resource settings, while AI and telemedicine represent emerging solutions for broader contexts. However, persistent system inequities continue to constrain universal benefit, highlighting the need for scalable, resource-appropriate strategies in global stroke care. Equitable implementation strategies tailored to local capacity remain essential for improving outcomes across all regions.

## CONCLUSION

This systematic review of 33 studies published between 2003–2025 demonstrates that early recognition and efficient referral are consistently associated with improved outcomes in acute stroke care. Structured recognition tools, including FAST, ROSIER, CPSS, and NIHSS, showed high sensitivity but modest specificity, reflecting a trade-off between timely detection and false-positive risk. System-level referral interventions such as EMS prenotification, dispatcher-supported triage, and mobile stroke units reduced treatment delays and increased eligibility for reperfusion therapies. Emerging AI-assisted recognition approaches showed promising diagnostic performance but remain heterogeneous and require further validation.

## Supplementary Information



## Figures and Tables

**Figure:**
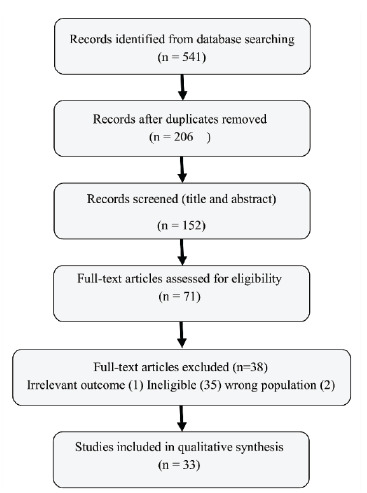
PRISMA 2020 flow diagram illustrates the process of identifying, screening, and assessing studies for eligibility in a systematic review of stroke recognition and referral pathways.

**Table 1:** Reported sensitivity and specificity of recognition tools across included studies in a systematic review of stroke recognition and referral pathways.

Tool / Model	First Author (Year)	Country	Sensitivity (%)	Specificity (%)	Notes
FAST	Harbison (2003)	UK	86	60	Prospective cohort; CT-confirmed
FAST	Saberian (2021)	Iran	94.8	55.1	MRI as gold standard
FAST-ED	Nasr-Esfahani (2021)	Iran	88.0 (≥2 cutoff)	57.5 (≥2 cutoff)	MRI gold standard; alternative cutoffs reported
PreHAST	Karimi (2020)	Iran	93.2 (≥1)	46.5 (≥1)	MRI gold standard
ROSIER	Saberian (2021)	Iran	95.0	60.1	MRI as gold standard
LAPSS	Saberian (2021)	Iran	71.9	82.8	MRI as gold standard
CPSS	Saberian (2021)	Iran	95.0	54.3	MRI as gold standard
Med PACS	Saberian (2021)	Iran	95.7	50.6	MRI as gold standard
OPSS	Saberian (2021)	Iran	80.8	59.5	MRI as gold standard
MASS	Saberian (2021)	Iran	86.7	61.8	MRI as gold standard
AI (DeepStroke)	Cai (2022)	USA	78.61	51.61	ER-based; compared to clinician impression
AI (PPG ML model)	Goda (2025)	Multicenter	66	74	Diagnostic feasibility study
AI (ChatGPT-4V)	Kuzan (2025)	Türkiye	79.57	84.87	Retrospective MRI analysis
AI (Hemodynamic ML)	García-Terriza (2023)	Spain	98.0	97.8	Based on hemodynamic monitoring
DARE-PACE	Yiang (2022)	Taiwan	85.4 (NIHSS≥8)	97.7 (NIHSS≥8)	Registry-based; CTA-confirmed

This table summarizes study-level diagnostic accuracy metrics (sensitivity and specificity) for stroke recognition tools evaluated between 2003 and 2025. Data are presented as reported in the original studies. Due to heterogeneity in populations, settings, and tool application, formal pooling was not feasible, and results were synthesized narratively.

*FAST*, Face Arm Speech Time; *ED*, emergency department; *PreHAST*, Prehospital Acute Stroke Severity Tool; *LAPSS*, Los Angeles Prehospital Stroke Screen; *ROSIER*, Recognition of Stroke in the Emergency Room; *CPSS*, Cincinnati Prehospital Stroke Scale; *Med PACS*, Melbourne Prehospital Assessment for Code Stroke; *OPSS*, Ontario Pre-hospital Stroke Scale; *MASS*, Melbourne Ambulance Stroke Scale; *AI*, artificial intelligence; *DARE-PACE*, Rapid Assay Diagnostic for Acute Stroke Recognition; *CT*, computed tomography, *MRI*, magnetic resonance imaging.

**Table 2:** Summary of characteristics of 33 studies included in a systematic review of stroke recognition and referral pathways.

Category	Summary
Number of studies	33 studies (2003–2025)
Regions represented	Europe, North America, Middle East, Asia
Study designs	6 randomized controlled trials; 8 cohort studies; 5 case-control; 14 cross-sectional/diagnostic validation
Sample sizes	Range: < 100 to > 4,000 participants
Age range	Predominantly middle-aged to elderly adults
Sex distribution	Slight male predominance across most studies (52–60%)
Stroke types	Ischemic stroke most common; several included hemorrhagic stroke and TIA
Recognition tools	FAST (Face Arm Speech Time), ROSIER (Recognition of Stroke in the Emergency Room), NIHSS (National Institutes of Health Stroke Scale), PreHAST (Prehospital Acute Stroke Severity Tool), DeepStroke, and EMS protocols
Imaging	CT and/or MRI in nearly all studies
Key outcomes reported	Diagnostic accuracy (sensitivity, specificity, predictive values), referral times, thrombolysis/thrombectomy eligibility, mortality, disability, and functional independence

*CT*, computed tomography; *EMS*, emergency medical services; *MRI*, magnetic resonance imaging; *TIA*, transient ischemic attack.

**Table 3:** Characteristics of Included studies n a systematic review of stroke recognition and referral pathways.

First author	Year	Design	Age	Sex	Stroke type	Onset to recognition	Referral outcome
Behrndtz	2023	Multicenter, randomized, assessor-blinded clinical trial	Median 74 years	103/171 (60%) male	AIS: 104/171 (61%); ICH: 51/171 (30%); Mimics: 16/171 (9%)	Median onset-to-randomization: 51 min	Door-in-door-out at PSC: 67 min (IQR 58–82)
Berg	2023	Retrospective cohort study	Mean 67.4 years	163/290 (56%) male	Ischemic: 151/290 (52%); Hemorrhagic: 17/290 (6%); TIA: 120/290 (41%)	177/290 (61%) ≤4.5h; 67/290 (23%) >4.5h	116/290 (30%) received correct visitation with high-priority ambulance
Berglund	2021	Prospective, observational cohort	Median ~74 years	~5,259/9,722 (54%) male	Ischemic, hemorrhagic, and mimics (n not reported)	Median admission: ~210 min high priority vs ~240 min low priority	High priority associated with ↑ IVT and EVT use
Blauenfeldt	2023	Multicenter, randomized, sham-controlled clinical trial	Median 72 years	not reported	Ischemic and hemorrhagic stroke (n not reported)	All within ≤4h	Target: confirmed acute ischemic or hemorrhagic strokes
Cai	2022	Diagnostic validation study (ED dataset)	≤65: 120/221; ≥65: 101/221	108/221 (49%) male	Suspected strokes (final confirmed at CT/MRI)	Screening <6 min	Sensitivity 78.6% (174/221); Specificity 51.6% (114/221) vs ER clinicians
Colton	2020	Prospective observational cohort study	Mean ~64 years	~47% male	Spontaneous intracerebral hemorrhage (ICH)	Not specified	Stroke code activation improved timeliness of ED interventions
Dela Ossa	2022	Multicenter, population-based, cluster-randomized clinical trial	Mean 75 years	~56% male	Ischemic/TIA: 452/654 (69%); Intracranial hemorrhage: 150/654 (22.9%); Mimics: 50/654 (7.7%)	Median onset-to-arrival: Thrombectomy group 67 min (IQR 44–164) vs local 59 min (40–111)	422/654 (64.6%) of local patients transferred for thrombectomy
De Luca	2009	Cluster randomized controlled trial	≤80 years	EMS: 1,187/2,656 (44.6%) male; ER: 1147/2239 (51.2%) male	Confirmed ICD-9 strokes	Eligibility onset ≤6h	SU referral: 646/2,656 (24.4%) intervention vs 293/2,239 (13.1%) control
Denti	2017	Stepped-wedge cluster RCT	Mean 73 years	902/1,622 (55.6%) male	Ischemic: 1,175/1,622 (72.4%); Hemorrhagic: 114/1,622 (7%); TIA: 195/1,622 (12%); Others: 139/1,622 (8.6%)	Median ~2h 40m overall	Early arrival <2h: 635/1,622 (39.2%); no significant campaign effect
Dolmans	2019	Observational diagnostic validation study (MIND-TIA cohort)	Mean 67.7 years	114/210 (54.4%) male	TIA: 104/210 (49.5%); Minor stroke: 22/210 (10.5%); Alternative diagnoses: 80/210 (38.1%)	All assessed ≤72h	128/210 (61%) confirmed as TIA/minor stroke; 82/210 (39%) alternative diagnoses
Ebinger	2015	Randomized-week clinical trial (STEMO)	≥18 years (exact mean not given)	n not reported	AIS confirmed in 518	Mean alarm-to-treatment: 51.8 min STEMO vs 76.3 min control	IVT: 171/518 (33%) STEMO vs 108/518 (21%) control
Esfahani	2021	Prospective diagnostic accuracy study	Mean 67.9 years	184/314 (58.6%) male	AIS confirmed: 274/314 (87.3%)	Onset to ED: 6.7h stroke vs 3.7h non-stroke	274/314 (87.3%) confirmed AIS
Goda	2025	Diagnostic feasibility study (ML-based)	Median 69.5 years (IQR 61–77.3)	57/88 (65%) male	LVO: 25/88 (28%); Non-LVO: 36/88 (41%); Mimics: 27/88 (31%)	PPG screening: 30 sec	Sensitivity 66% (58/88); Specificity 74% (65/88); AUROC 0.77
Govindarajan	2015	Cross-sectional, EMS–hospital linked registry	≥80 years	382/855 (44.7%) male	Mixed stroke codes (ICD-9: 430–438)	Not reported	IV tPA: 34/855 (4%)
Gude	2023	National observational cohort	Median ~73 years	~2,056/3,546 (58%) male	AIS: 3,546/3,546 (100%)	All ≤3h	Recognized: 3,042/3,546 (85.8%) admitted vs 2,641/3,546 (74.5%) unrecognized
Guterud	2023	Randomized controlled trial	~70 years	not reported	AIS + ICH	≤4.5h	More rapid CT and IVT in modified tool group
Harbison	2003	Prospective observational cohort	~72 years	148/302 (49%) male	Ischemic (majority), ICH, mimics	Median 3h	↑ IVT eligibility in early recognition
Helwig	2019	Randomized, multicenter, parallel-group	Mean 74 years	not reported	OPM: AIS 121/165 (73.6%); ICH 25/165 (15.1%); TIA 12/165 (7.5%); MSU: AIS 107/210 (50.8%); ICH 27/210 (12.7%); TIA 57/210 (27%)	≤8h or wake-up stroke	Secondary transfers: OPM 68/165 (41.2%) vs MSU 0/210 (0%)
Karimi	2020	Cross-sectional diagnostic accuracy	Mean 67.1 years	462/804 (57.5%) male	AIS: 562/804 (69.8%) confirmed by MRI	ED arrival	Sensitivity 93.2% (750/805); Specificity 46.5% (117/252)
Karliński	2022	Retrospective observational	~71 years	354/690 (51.3%) male	CVA confirmed: 690/690	ED referrals 2014	Correct prehospital dx: 469/690 (68%)
Karliński	2015	Prospective observational	73 years	192/732 (26.2%) male	Ischemic 556/732 (75.9%); TIA 100/732 (13.6%); ICH 77/732 (10.5%)	Not specified	Misdiagnoses: 271/732 (37%); PCP/outpatient 51.6% vs ambulance 32.5%
Kuzan	2024	Retrospective diagnostic accuracy	>18 years	n not reported	AIS confirmed: 266/530 (50.2%)	Imaging ≤24h	TIAs excluded
Li	2024	Open-label multicenter RCT	Mean 70 years	~281/455 (61.7%) male	Ischemic: 280/455 (61.5%); ICH 68/455 (15%); Mimics 107/455 (23.5%)	Median 61 min (IQR 41–93)	Early BP reduction achieved
Manawadu	2014	Registry-based observational cohort	~74 years	214/420 (51%) male	Ischemic/TIA majority	336/420 (80%) ≤3h	Early referrals: IVT 74/77 (96%) vs 31/103 (30%)
Oostema	2015	Prospective registry study	Mean 78 years	122/296 (41.2%) male	AIS: 186/441 (42.2%); TIA: 78/441 (17.7%); Hemorrhagic excluded	90/186 AIS (48.4%) ≤120 min	EMS-recognized: shorter CT time (34.6 vs 84.7 min)
Price	2020	Multicenter cluster RCT	Mean 74.7 years	not reported	PASTA: 409/499 (82%) AIS; SC: 607/714 (85% AIS)	Paramedic assessment ≤4h	IVT: 197/499 (39.4%) PASTA vs 319/714 (44.7%) SC
Saberian	2021	Multicenter diagnostic accuracy	Mean 66.9 years	463/926 (57.5%) male	AIS confirmed: 562/804 (69.8%)	ED arrival	Sensitivity/Specificity per PreHAST cut-off
Saver	2015	Multicenter, randomized phase 3 trial	Mean 69 years	not reported	Ischemic 278/380 (73.3%); ICH 87/380 (22.8%); Mimics 15/380 (3.9%)	Median 45 min; 282/380 (74.3%) treated ≤1h	Rapid IVT; ~24% ICH/mimic
Sundström	2017	Retrospective multicenter	Mean ~78 years	159/352 (45%) male	ICH 68/352 (19%); Cerebral infarction 221/352 (63%); Other 63/352 (18%)	Median system delay (EMS call → CT)	Priority 1: faster CT and IVT use
Terriza	2023	Machine learning–based diagnostic	Not reported	n not reported	ML differentiated ischemic vs hemorrhagic	Not reported	Improved early triage
Van Den Berg	2022	Ambulance-based RCT	Mean ~72 years	GTN arm: 58% male; Control: 47% male	Ischemic 148/236 (63%); ICH 39/236 (16%); TIA 27/236 (11%); Mimic 22/236 (9%)	Median 53–71 min	All transferred; some later excluded
Yiang	2022	Retrospective registry	Not reported	n not reported	AIS: 98/147 (67%); Minor strokes 49/147 (33%)	Door-to-CT 13.4 ± 1.8 min; Door-to-CTA 75.5 ± 44.5 min	Avoided 98/147 (66.6%) unnecessary CTA

Characteristics of included studies assessing stroke recognition, referral outcomes, and diagnostic accuracy. Data are summarized by first author, publication year, study design, participant demographics, stroke type, onset-to-recognition time, and referral outcomes.

*ICH*, intracranial hemorrhage; AIS, abbreviated injury scale; *TIA*, transient ischemic attack; *PSC*, Primary Stroke Center; *IVT*, intravenous therapy; *EVT*, Endovascular Thrombectomy; *CT*, computed tomography; *MRI*, magnetic resonance imaging; *ER*, emergency room; *ED*, emergency department.

**Table 4:** Risk-of-bias assessment of the 33 included studies in a systematic review of stroke recognition and referral pathways.

First author	Year	Study design	Study size	Risk-of-bias judgment
van den Berg	2022	RCT	236	Some concerns
Wireklint	2017	Cohort	352	Moderate
De Luca	2009	Cluster RCT	4,895	Low
Cai	2022	Diagnostic	221	Moderate
Gude	2023	Cohort	3,546	Low
Berg	2023	Cohort	290	Moderate
Saberian	2021	Diagnostic	926	Low

**Table 5:** Risk of bias assessment of the 33 studies included in a systematic review of stroke recognition and referral pathways.

First author	Year	Study design	Risk-of-bias judgment
Behrndtz	2023	Multicenter, randomized, assessor-blinded clinical trial	Unclear
Berg	2023	Retrospective cohort study (registry and recorded emergency calls)	Unclear
Berglund	2021	Prospective, observational cohort study	Unclear
Blauenfeldt	2023	Multicenter, randomized, patient- and outcome-assessor-blinded, sham-controlled clinical trial	Unclear
Cai	2022	Diagnostic validation study using cross-validation and hold-out testing on ER patient dataset	Some concerns
Colton	2020	Prospective observational cohort study	Unclear
Pérez de la Ossa	2022	Multicenter, population-based, spatial-temporal cluster-randomized clinical trial	Unclear
De Luca	2009	Cluster randomised controlled trial	Low
Denti	2017	Stepped-wedge cluster randomized controlled trial	Unclear
Dolmans	2019	Observational diagnostic validation study (using MIND-TIA cohort)	Moderate
Ebinger	2015	Randomized-week clinical trial (open-label)	Unclear
Nasr-Esfahani	2021	Prospective diagnostic accuracy study	Unclear
Goda	2025	Diagnostic feasibility study using machine learning	Some concerns
Govindarajan	2015	Cross-sectional observational study using probabilistic linkage of EMS and hospital records	Unclear
Gude	2023	National observational cohort study	
Guterud	2023	Randomized controlled trial	Unclear
Harbison	2003	Prospective observational cohort study	Unclear
Helwig	2019	Prospective, randomized, multicenter, parallel-group clinical trial	Unclear
Karimi	2020	Cross-sectional multicenter diagnostic accuracy study	Unclear
Karliński	2022	Retrospective observational study	Unclear
Karliński	2015	Prospective observational study	Unclear
Kuzan	2025	Retrospective diagnostic accuracy study	Unclear
Li	2024	Open-label, multicenter, randomized clinical trial with blinded outcome assessment	Unclear
Manawadu	2014	Prospective registry-based observational cohort study	Unclear
Oostema	2015	Prospective observational registry study	Unclear
Price	2020	Multicenter cluster randomized clinical trial	Unclear
Saberian	2021	Multicenter diagnostic accuracy study	Unclear
Saver	2015	Multicenter, randomized, double-blind, placebo-controlled phase 3 trial	Unclear
Fakhraldeen	2015	randomized clinical trial	Unclear
Sundström	2017	Observational retrospective multicenter study	Moderate
Terriza	2023	Machine learning-based diagnostic and predictive modeling study	Unclear
Van Den Berg	2022	Randomised controlled trial (Phase 3, multicenter, ambulance-based, open-label, blinded endpoint)	Unclear
Yiang	2022	Retrospective registry-based diagnostic accuracy study	Unclear

*EMS*, emergency medical services; *MIND-TIA*, Multicenter Imaging Study for Transient Ischemic Attack.

**Table 6:** Diagnostic accuracy of recognition tools for acute stroke in primary and emergency care settings, as reported in a systematic review of stroke recognition and referral pathways.

First author	Year	Recognition tool(s)	Diagnostic accuracy metrics reported?
Behrndtz	2023	PASS	No
Berglund	2021	Symptom-based EMS triage	No
Blauenfeldt	2023	PreSS	No
Cai	2022	AI-based DeepStroke	Yes
De Luca	2009	CPSS (EMS), NIHSS (ED)	No
Denti	2017	Public education campaign	No
Ebinger	2014	Mobile stroke unit (STEMO)	No
Goda	2025	PPG ML Model; Hunter-8	Yes
Gude	2023	Danish Index Dispatcher	No
Harbison	2003	FAST	Yes
Karimi	2020	PreHAST	Yes
Karliński	2022	EMS physician/paramedic judgment	No
Karliński	2015	Physician judgment	No
Kuzan	2025	ChatGPT-4V MRI interpretation	Yes
Nasr-Esfahani	2021	FAST-ED	Yes
Parsberg Berg	2023	Danish Index Dispatcher	No
Price	2020	FAST (PASTA trial)	No
Saberian	2021	ROSIER, FAST, CPSS, LAPSS, Med PACS, OPSS, MASS, PreHAST	Yes
Terriza	2023	ML hemodynamic signals	Yes
Van Den Berg	2022	FAST	No
Sundström	2017	Dispatcher Medical Index, RETTS, RLS-85	No
Yiang	2022	DARE-PACE + NIHSS thresholds	Yes

Tools range from prehospital screening methods to advanced technologies (AI-based DeepStroke, ML hemodynamic signals, ChatGPT-4V MRI interpretation), highlighting variability in diagnostic validation across emergency care contexts.

**Table 7:** Referral patterns and prehospital interventions in a systematic review of stroke recognition and referral pathways.

First author	Referral intervention	Key outcome
Sundström	RETTS + Dispatcher Index	Improved priority assignment; reduced under-triage
Gude	Danish Index Dispatcher	Shorter onset-to-door times; increased thrombolysis eligibility
Ebinger	Mobile Stroke Unit (STEMO)	Reduced time to CT; increased reperfusion rates
Price	PASTA trial (paramedic training)	No significant effect on delay
Karimi	PreHAST scale in EMS	increased detection of LVO; expedited referral
Berg	Danish Index Dispatcher	Improved EMS prioritization; shorter delays
Behrndtz	PASS scale (prehospital triage)	Moderate improvement in LVO triage

*CT*, computed tomography; *EMS*, Emergency Medical Services; *LVO*, large vessel occlusion; *PASS*, Prehospital Acute Stroke Severity Scale; *RETTS*, Rapid Emergency Triage and Treatment System; *STEMO*, Stroke-Einsatz-mobile stroke unit.

**Table 8:** Subgroup analysis of diagnostic accuracy and referral patterns in acute stroke recognition from 33 studies included in a systematic review of stroke recognition and referral pathways.

Subgroup	No. of Studies	Key Tools	Sensitivity Range	Specificity Range	Main Finding
Primary Care	9	FAST, PreHAST	70–90%	45–60%	Delays > 4.5 hours common
Emergency Care	24	NIHSS, AI tools	80–95%	55–85%	Higher reperfusion eligibility
Europe	14	FAST, Dispatcher Index	82–95%	55–75%	Faster referral with dispatcher protocols
Asia/Middle East	12	PreHAST, ROSIER	70–88%	50–65%	Limited imaging and EMS capacity
North America	7	AI-based DeepStroke, ChatGPT-4V	75–85%	70–80%	Early AI adoption; promising specificity

*AI*, artificial intelligence; *EMS*, emergency medical services; *FAST*, Face Arm Speech Time; *NIHSS*, National Institutes of Health Stroke Scale; *PreHAST*, Prehospital Acute Stroke Severity Tool; *ROSIER*, Recognition of Stroke in the Emergency Room.
