# Supplementary material for: Early Recognition and Referral of Acute Stroke in Primary and Emergency Care: A Systematic Review
Source: West J Emerg Med. 2026 Jan 24;27(3):804–18. doi: 10.5811/westjem.50827 (PMC13246193; doi:10.5811/westjem.50827)
Supplement: Supplementary file 1 [file wjem-27-804-s001.docx]

**Appendix.** Full Search Strategies for Each Database

**PubMed (MEDLINE)**

- Search performed using MeSH terms and free-text keywords:

("Stroke"[MeSH] OR "Acute Ischemic Stroke"[MeSH] OR stroke OR "acute stroke"

OR "ischemic stroke" OR "hemorrhagic stroke")

AND

("Diagnosis"[MeSH] OR "Diagnostic Accuracy" OR "early recognition"

OR "stroke recognition" OR "stroke screening")

AND

("Primary Health Care"[MeSH] OR "primary care" OR "general practice"

OR "Emergency Medical Services"[MeSH] OR "emergency department"

OR "emergency care")

AND

("Referral and Consultation"[MeSH] OR referral OR transfer OR triage)

AND

("Treatment Outcome"[MeSH] OR outcome OR mortality OR disability

OR "functional outcome")

Filters applied: Humans; English; Publication years 2003–2025

**Embase**

- Search conducted using Emtree terms and keywords:

('stroke'/exp OR 'acute ischemic stroke' OR 'hemorrhagic stroke')

AND

('diagnostic accuracy' OR 'early recognition' OR 'stroke recognition')

AND

('primary care' OR 'general practice' OR 'emergency care' OR 'emergency department')

AND

('referral' OR 'transfer' OR 'triage')

AND

('treatment outcome' OR mortality OR disability)

Limits: English language; Humans; 2003–2025

**Scopus**

TITLE-ABS-KEY(stroke OR "acute stroke" OR "ischemic stroke" OR "hemorrhagic stroke")

AND

TITLE-ABS-KEY("diagnostic accuracy" OR "early recognition" OR "stroke recognition")

AND

TITLE-ABS-KEY("primary care" OR "emergency care" OR "emergency department")

AND

TITLE-ABS-KEY(referral OR transfer OR triage)

AND

TITLE-ABS-KEY(outcome OR mortality OR disability)

Limits: English; 2003–2025

**Web of Science**

TS=(stroke OR "acute stroke" OR "ischemic stroke" OR "hemorrhagic stroke")

AND

TS=("diagnostic accuracy" OR "early recognition" OR "stroke recognition")

AND

TS=("primary care" OR "emergency care" OR "emergency department")

AND

TS=(referral OR transfer OR triage)

AND

TS=(outcome OR mortality OR disability)

Timespan: 2003–2025; Language: English

**Cochrane Library**

(stroke OR "acute stroke" OR "ischemic stroke" OR "hemorrhagic stroke")

AND

("early recognition" OR "diagnostic accuracy")

AND

("primary care" OR "emergency care")

AND

(referral OR transfer OR triage)

AND

(outcome OR mortality OR disability)

Publication years: 2003–2025

**Grey Literature**

- Additional sources included:
- World Health Organization (WHO) reports
- Major stroke organizations (e.g., American Stroke Association, European Stroke Organisation)
- Searches were conducted using relevant keywords related to stroke recognition, referral pathways, and outcomes.
